# Exosomal tRF-Leu-AAG-001 derived from mast cell as a potential non-invasive diagnostic biomarker for endometriosis

**DOI:** 10.1186/s12905-022-01827-6

**Published:** 2022-06-25

**Authors:** Yingxue Li, Shuling Cui, Zemin Xu, Yanping Zhang, Tao Wu, Jing Zhang, Yichen Chen

**Affiliations:** 1 Ningbo Women&Children’s Hospital, Ningbo, China; 2 The Affiliated Hospital of Medical School of Ningbo University, Ningbo, China; 3 Ningbo Institute of Medical Sciences, Ningbo, China; 4 Ningbo University, Ningbo, China; 5 Addiction Research of Zhejiang Province, Ningbo Kangning Hospital, School of Medicine, Ningbo University, Ningbo, China

**Keywords:** Endometriosis, Exosomes, Leucorrhea, Transfer RNA-derived fragment, Diagnostic biomarker

## Abstract

**Background:**

The diagnosis of endometriosis (EMs) is still based on laparoscopic observation. This study tries to verify whether exosomal tRNA-derived fragments (tRFs) in leucorrhea can be used as non-invasive diagnostic markers.

**Methods:**

Endometrial tissues and leucorrhea were sampled from women hospitalized in Ningbo University Affiliated Hospital from January 2021 to July 2021 with (n = 26) and without endometriosis (n = 25). Exosomes were isolated from samples by differential centrifugation. The small RNA sequencing was performed to detect the exosomal tRNA halves (tiRNAs)&tRFs. RNA probe and immunofluorescence antibody were used to localize the origin of tRFs. From mast cell lines infected with tRF-Leu-AAG-001 siRNA, we observed the change in vascular capacity and expression of inflammatory factors. The specificity and sensitivity tRF were determined by receiver operating characteristic analyses.

**Results:**

63 up-regulated and 45 down-regulated tRFs&tiRNAs were identified in ectopic exosomes. We selected tRF-Leu-AAG-001 as a candidate marker through KEGG pathway enrichment and PCR verification. We found that mast cells highly expressed tRF-Leu-AAG-001 in ectopic foci by immunofluorescence staining. We used siRNA to silenced tRF-Leu-AAG-001 expression in luva, qPCR analysis showed IL-6, IL-10, IL-1β, and TNF-α were significantly decreased. Meanwhile, tRF-Leu-AAG-001 siRNA dramatically reduced the angiogenic ability of luva. Finally, we examined the expression of exosomal tRF-Leu-AAG-001 in the leucorrhea. It was found exosomal tRF-Leu-AAG-001 had high specificity and sensitivity for predicting the occurrence of ectopic disease.

**Conclusions:**

Exosomal tRF-Leu-AAG-001 derived from mast cells in ectopic foci might promote inflammation and angiogenesis. Meanwhile, leucorrhea exosomal tRF-Leu-AAG-001 could be a potential diagnostic biomarker for endometriosis.

**Supplementary Information:**

The online version contains supplementary material available at 10.1186/s12905-022-01827-6.

## Introduction

Endometriosis (EMs) is a common hormone-dependent disease characterized by the growth of endometrial tissue (glands and stroma) outside the uterine cavity and myometrium. According to the location of the ectopic lesion, the type of endometriosis can be categorized as superficial peritoneal lesions, ovarian cysts (endometriomas), deep endometriosis [1] and some rare extra-pelvic lesions [2]. EMs can cause dysmenorrhea, infertility, abdominal mass, chronic pelvic pain, and acute abdominal pain, affecting about 10% (190 million women worldwide) of women of reproductive age [3], even affecting some postmenopausal women [4]. Although multiple theories have been proposed, the pathogenesis of endometriosis remains controversial: retrograde menstruation [5], inflammation [6], immunity [7] and metabolic factors [8] have all been advocated to explain the complex mechanisms behind the development of Ems. In addition, recent studies have found that abnormal alterations in macrophage counts [9], microbial levels [10], intestinal permeability [11] and epigenetic expression profiles [12] in Ems patients, which affect the local and systemic inflammatory environment, may also be important in the pathogenesis of Ems.Due to the lack of understanding of exact etiology of EMs, currently available clinical treatment and diagnostic approaches are still ineffective for most patients, which is significantly affecting patients' quality of life [13]. Therefore, further understanding of the pathogenesis, development of new useful biomarkers, and timely diagnosis and treatment of the disease remain the current priorities.

tRNA-derived small RNAs(tsRNAs) are the new type of small non-coding RNAs derived from tRNA, which are about 18–40 nucleotides in length. tsRNAs can be divided into two main types: tiRNAs (tRNA halves) and tRFs (tRNA-derived fragments) [14]. According to studies, the function of tRFs such as miRNAs is considered an essential regulator of various diseases like cancer [15], acquired metabolic diseases [16], infectious diseases [17] and neurodegenerative diseases [18]. Moreover, an increasing number of research is starting to show that exosomal tRFs are the potential disease modulators [19] and circulating diagnostic markers [20].

Exosomes are small extracellular vesicles (EVs) with a 30-150 nm diameter secreted by living cells [21]. They are widely present in various body fluids such as blood, urine, saliva and breast milk, as well as in tissues and intercellular spaces [22]. Exosomes can mediate cell–cell communication by transmitting regulatory molecules and genetic information (lipids, proteins, DNA and complex RNA) [23]. Numerous reports have suggested that exosomes play important regulatory roles in the development of endometriosis. For instance, exosomal lncRNAs and miRNAs are able to accelerate blood vessel regeneration [24] and even cause infertility [25]. However, it is rarely reported the role of exosomal tRFs in EMs.

In this study, we isolated exosomes from ectopic tissues and sequenced tiRNA&tRFs to screen out the specifically expressed tRF-Leu-AAG-001 in ectopic tissues. We assessed the origin and biological function of tRF-Leu-AAG-001. Finally, the expression of exosomal tRF-Leu-AAG-001 was evaluated in the leucorrhea of EMs patients. The aim of our study is to find a novel biological marker for the non-invasive diagnosis of endometriosis.

## Materials and methods

### Ethics approval and consent to participate

The written informed consent of each patient participating in the study was obtained. The study protocol and informed consent were approved by the ethics committee of the Affiliated Hospital of Medical School of Ningbo University. All of the methods were carried out in accordance with the Declaration of Helsinki.

### Sample collection

All samples (normal/ectopic endometrial tissues and leucorrhea) were collected in the Affiliated Hospital of Medical School of Ningbo University from March 2020 to March 2021. A total of 51 females were enrolled in our study. Among all patients, 26 patients who were diagnosed with EMS through laparoscopy and histopathological examination served as the control group. The remaining 25 patients with non-endometriosis who were admitted to the hospital during the same period included as the control group. Inclusion criteria: 1. No history of treatment with hormones or antibiotics within three months before laparoscopic surgery; 2. No hepatitis, tuberculosis, tumors and other diseases. Exclusion criteria: 1. Treated with hormones and antibiotics recently; 2. With serious organic diseases; 3. Combined with other gynecological diseases such as inflammation of the reproductive system and tumors. (The general information of the enrolled patients was shown in Additional file 1: Table S1). All subjects who had regular menstrual cycles were women of childbearing age who were in non-menstrual period three days before the sample collection. Mast cell line-Luva was a generous gift from a laboratory at Zhejiang University.

### Exosomes isolation from tissues and leucorrhea

We used differential centrifugation to extract exosomes from tissues and leucorrhea. Briefly: ectopic tissue was disaggregated into a single cell suspension with type IV collagenase (Solarbio, China). Leucorrhea was diluted with PBS to make a mixed solution. The supernatant and leucorrhea solution were centrifuged at 4 °C with a high-speed centrifuge (Thermo, USA) at 500 g for 10 min to remove living cells, 2000 g for 10 min to remove dead cells, and 10,000 g for 20 min to eliminate the cell debris. Every step was repeated twice. The supernatant was then centrifuged at 100,000 g twice with ultracentrifuge (Beckman, USA) for 70 min each time. The exosomes were resuspended or lysed with different reagents for subsequent experiments.

### Exosomal size identification

Transmission electron microscopy (TEM) was used to identify the size of exosomes. Briefly, the exosome was dropped on the copper net for 5 min at room temperature. 3% phosphotungstic acid solution stained the nanoparticles. Then, exosomes were analyzed with a transmission electron microscope (Hitachi H-7650). The diameter distribution of exosomes was examined by nanoparticle Tracking Analysis (NTA) (Malvern NanoSight NS500).

### Immunoblotting for exosomal markers

Exosomes were lysed with a RIPA buffer, resuspended in the loading buffer, boiled at 95 °C for 5 min, and then electrophoresed on SDS-PAGE. Proteins were transferred to polyvinylidene fluoride membrane, which was blocked with 5% non-fat dry milk in TBST. Immunodetection was performed with anti-HSP70 antibody(1:1000, Proteintech, China), anti-Flotillin-1 antibody (1:1000, Proteintech, China), anti-CD63 antibody (1:1000, Proteintech, China) and anti-calnexin antibody (1:1000, Proteintech, China) at a dilution of 1:1000 followed by incubation at 4 °C overnight. The next day, protein was incubated with appropriate HRP-conjugated secondary antibody (1:5,000, Abcam, USA). Bands were revealed using ECL Plus and then imaged on the electrophoresis gel imaging analysis system (D-Digital, USA) to analyze.

### Library construction and small RNA sequencing

ExoRNA was extracted with Trizol reagent (Invitrogen, USA), and purified RNA was sent to Aksomics Biological Engineering Co., Ltd. (Shanghai, China) for performing tRFs & tiRNAs sequencing analysis. The brief steps were as follows: agarose gel electrophoresis was used to detect the integrity of the total RNA sample, and NanoDrop ND-1000 quantitative analyzer (thermos, USA) quantified RNA concentration. TRF&tiRNA-seq library preparation includes: (1) 0.3′-adapter ligation; (2) 5′-adapter ligation; (3) cDNA synthesis; (4) PCR amplification; (5) size selection of 134-160 bp PCR amplified fragments (corresponding to ~ 14-40nt small RNA). The library was quantitatively analyzed with Agilent 2100 bioanalyzer. According to the quantitative results, the library was mixed in equal amounts. The DNA fragments in the mixed library were denatured with 0.1 M NaOH to generate single-stranded DNA molecules, which were loaded onto the kit at a concentration of 1.8 pM. According to the manufacturer's instructions, the NextSeq 500/550 V2 kit (#FC-404–2005, Illumina) was used for sequencing with the NextSeq system. R package edgeR software was used to screen the differentially expressed TRFs and tiRNAs based on the count value.

### Quantitative real-time polymerase chain reaction (qRT-PCR)

According to the manufacturer’s instructions, total RNAs were extracted from purified exosomes and cultured cells using Trizol reagent (Invitrogen, USA). The extracted RNA was stored at − 80 °C. The cDNAs were synthesized by using a reverse transcription kit, according to manufacturer's instructions (CWbio, Beijing, China). qRT-PCR for cellular and exosomal RNA, including tRF-Leu-AAG-001, tRF-Leu-TAG-015, IL-6, IL-10, IL-1β, TNF-α and GAPDH, were performed using RT-PCR quantitation kit (CWBio, Beijing, China). Briefly, after an initial denaturation step at 95 °C for 10 min, the amplifications were carried out with 40 cycles at a melting temperature of 95 °C for 15 s, and an annealing temperature of 60 °C for 30 s. The relative expression levels of mRNAs were calculated with 2–ΔCt method. PCR productions of tRF-Leu-AAG-001, tRF-Leu-TAG-015 were tested by 3% agarose. The sequences of the specific primers were presented in Table 1.

**Table 1:** The primer sequences of all genes

Gene name	Forward(5′ to 3′)	Reverse(5′ to 3′)
tRF-leu-AAG-001	ATCCCACCGCTGCCACCA	
tRF-leu-TAG-015	ATCCCACCACTGCCACCA	
IL-6	ACTCACCTCTTCAGAACGAATTG	CCATCTTTGGAAGGTTCAGGTTG
IL-10	GACTTTAAGGGTTACCTGGGTTG	TCACATGCGCCTTGATGTCTG
IL-1β	ATGATGGCTTATTACAGTGGCAA	GTCGGAGATTCGTAGCTGGA
TNF-α	CCTCTCTCTAATCAGCCCTCTG	GAGGACCTGGGAGTAGATGAG
GAPDH	GAAGGTGAAGGTCGGAGT	GAAGATGGTGATGGGATTTC
U6	CGCTTCGGCAGCACATATAC	TTCACGAATTTGCGTGTCAT

### 3D cell culture

Ectopic tissues were digested into the single-cell suspension with type IV collagenase (Solarbio, China). After centrifugation to pellet the cells, NanoShuttle (50 μl, Greiner bio-one Co., Germany) was added to the cell suspension, and incubated the cell-nano mix suspension was incubated at 37 °C for 1 h. After centrifugation to remove the supernatant, the number of cells was adjusted to 8*10^4^/150ul with the medium mix. The cells were inoculated into a 96 well microplate (cell-repellent surface, Greiner bio-one Co., Germany). Then we hold the microplate on a magnetic driver (Greiner bio-one Co., Germany). The cell balls were placed in a 37 °C, 5% cell incubator and incubated for 15 min, and then the magnetic driver was removed.

### Fluorescence positioning

We used immunofluorescence and RNA fluorescence probes for co-localization of tRF-Leu-AAG-001 and mast cells. We purchased the Cy3-labeled tRF-Leu-AAG-001 fluorescence probe from Ruibo Biotech, and purchased the mast cell marker: anti-CD117-FITC antibody from Thermo Fisher. Briefly: 3D primary ectopic cells were inoculated in 96-well plate for 1 h, Cy3-labeled tRF-Leu-AAG-001 fluorescence probe was added and incubated overnight at 37 °C. The next day, cell balls were washed with PBS for 5 min, protected from light, three times, then added anti-CD117-FITC antibody and incubated at 37 °C for 1 h. Aspirated the secondary antibody and washed with PBS in the dark. Finally, added DAPI solution at room temperature for 5 min, photograph the fluorescence with Olympus confocal microscope.

### knockdown of tRF-Leu-AAG-001 by Small interfering RNA

tRF-Leu-AAG-001 siRNA and negative control (NC) were designed and compounded by Sangon Biotech. Luva was seeded into 6-well plates, and then they were transfected of siRNA by using Lipofectamine 2000(Invitrogen, USA). After 24 h, cells were digested and transferred to T75 culture flask, and we collected the cell supernatant for exosomes isolation at 24 h and 48 h.

### Tube formation assay

The 96-well plate was pre-coated by Matrigel. Before the test, human umbilical vein endothelial cells (HUVECs) were cultured with ECM medium containing 100 × growth factor and 5%FBS for 24 h. HUVECs were co-cultured with four groups for 24 h, including luva group, luva treated with tRF-Leu-AAG-001 siRNA group, exosomes derived from luva group, and exosomes treated with tRF-Leu-AAG-001 siRNA group. After treatment, HUVECs were added to 96 wells with 2.5*10^4^ cells per well. The vascularization phenomenon was observed under the Olympus microscope. ImageJ software was used to measure blood vessel nodes and capillary length.

### Immunofluorescence

This assay was performed to identify the internalization of the exosomes from mast cells into HUVECs. Briefly, isolated exosomes were re-suspended in 200 ul of PBS in a 1.5 ml microcentrifuge tube. Then mast cell-derived exosomes were labeled according to the instructions using the PKH67 Green Fluorescent Cell Linker Mini Kit(Umibio Science and Techology,China) and incubated at 37 °C for 1 h without shaking. Labeled exosomes were centrifuged at 10000 g for 70 min, and the supernatant was carefully filtered with a 0.22-μm filter. PHK67-labeled exosomes were then co-cultured with HUVECs for 24 h in a 6-well plate. The cells were then prepared for immunofluorescence analysis, and the internalization of exosomes was subsequently observed under a Confocal laser scanning microscope (LEICA TCS SP8,Germany).

### Statistical analysis

The experimental data were statistically analyzed using GraphPadPrism8.0 (GraphPad Software, USA) and SPSS software (version 21.0; IBM, Armonk, NY, USA). Measurement data were expressed as mean ± standard deviation (SD). Statistical comparisons between the two groups were performed using a Two-tailed Student's t-test, and multiple comparisons were performed using a One-Way Analysis of Variance (ANOVA). *P* value < 0.05 indicates statistical significance.

## Results

### Identification of ectopic tissue and leucorrhea exosomes

**Fig. 1:**
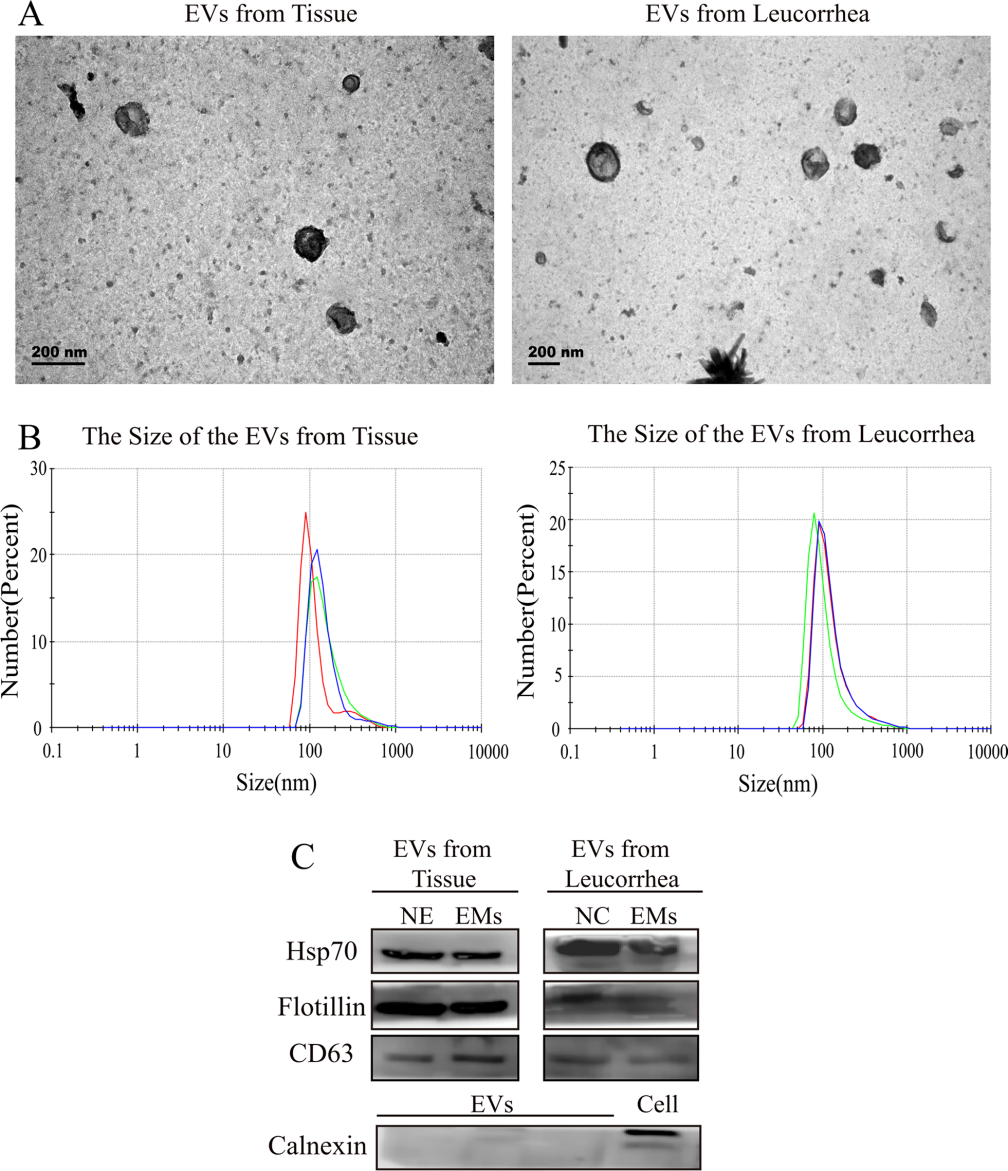
Identification of isolated EVs in tissues and leucorrhea. **A** The morphology of EVs was analyzed by TEM (< 200 nm). **B** The diameter distribution of EVs was analyzed with a nanoparticle analyzer. **C** Western blot analysis of exosomal marker protein Flotillion 1, HSP70, CD63, Calnexin. Western blot results showed HSP70, flotillion 1 and CD63, three well-known protein markers, were enriched in exosomes from ectopic tissue and leucorrhea but were undetectable in the cells.While the exosomal negative marker protein, calnexin, was expressed in cells

To identify the characteristics of exosomes derived from different sources of samples, we used transmission electron microscopy (TME) and nanoparticle tracking analysis (NTA) to observe the size of exosomes. Western blotting was used to clarify the protein markers of extracellular vesicles. Exosomes showed the typical cup-shaped structure with an obvious membrane under TME (Fig. 1A). The average diameters of EVs particles measured by NTA were 100 nm ± 30 nm (Fig. 1B). WB results showed exosomal positive marker proteins, flotillion 1, HSP70 and CD63 were expressed in exosomes, which were purified from ectopic tissue and leucorrhea, while the exosomal negative marker protein, calnexin, was expressed in cells (Fig. 1C) (Additional file 3). (The original images of the immunoblots were shown in the Additional file 4: Figures).

### Study on the tRFs & tiRNAs profiles of exosomes in ectopic tissues

**Fig. 2:**
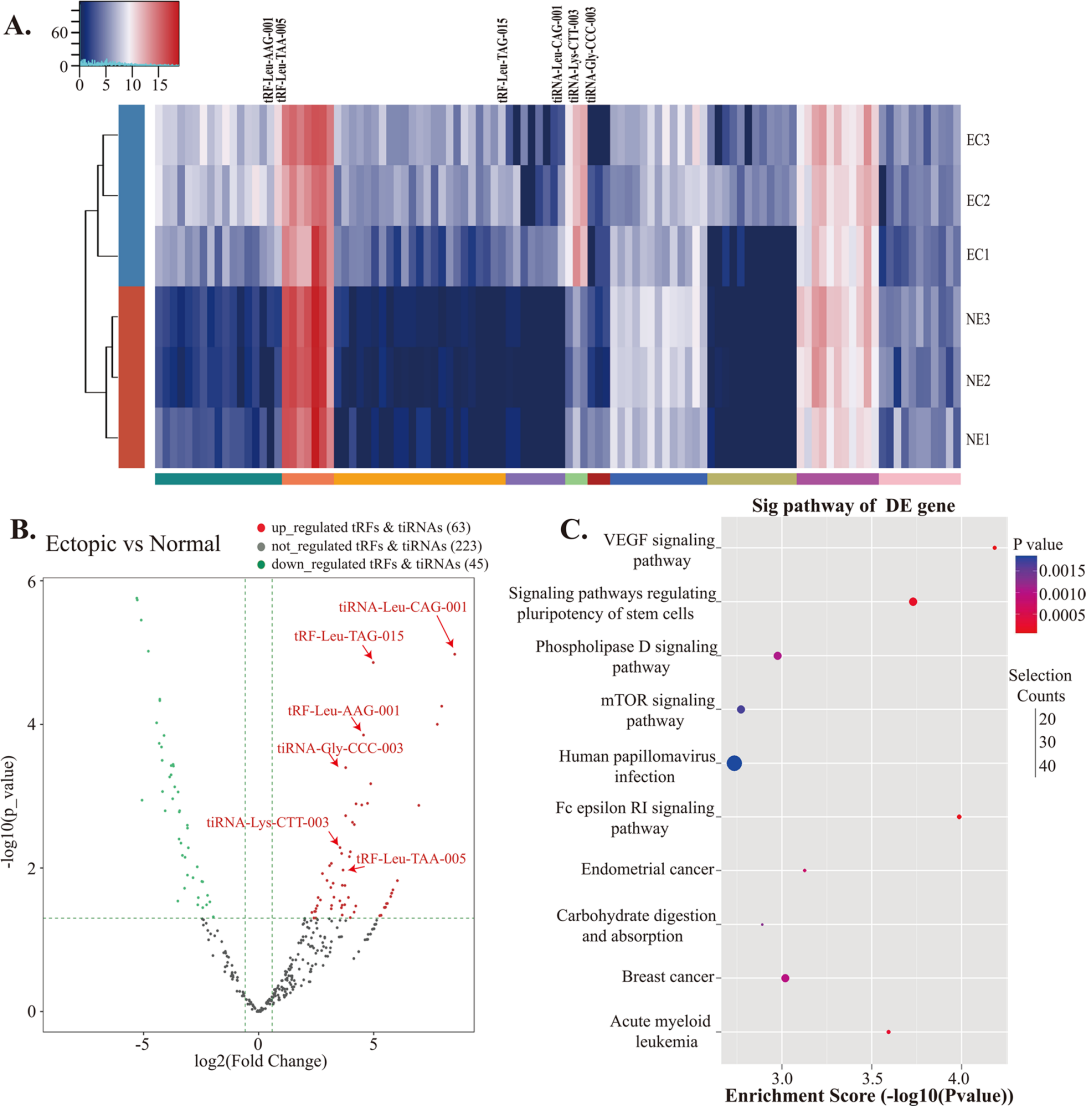
Identification of exosomal tRFs& tiRNAs related to EMs. **A** Generate heat map after hierarchical cluster analysis. The red (up-regulated) and green (down-regulated) tRFs & tiRNAs expression differences were statistically significant (*p < 0.05). **B** The volcano graph compares the fold change in the expression of tRFs & tiRNAs in exosomes from EMs patients and healthy controls. Red dots represent up-regulated tRFs & tiRNAs, and green dots represent down-regulated tRFs & tiRNAs. **C** Enrichment analysis of candidate target gene KEGG

Exosomal RNAs were extracted from ectopic tissue (n = 3) and normal endometrial tissues (n = 3). tRFs & tiRNAs sequencing was performed on the exosomal RNAs. By analyzing the original tRFs & tiRNAs expression profile data, 331 differential tRFs or tiRNAs were screened between the control and EMs groups (Fig. 2A). Based on the > 1.5-fold difference between the two groups, 108 tRFs or tiRNAs (63 up-regulated and 45 down-regulated) were selected (Fig. 2B). Next, we selected seven highly expressed tRFs&tiRNAs in ectopic exosomes to perform KEGG pathway analysis (Additional file 2: Table S2) and found that these specifically expressed tRF&tiRNAs were mainly enriched in ten pathways (Fig. 2C) [26], of which the VEGF signaling pathway and Fc epsilon IR signaling pathway were the most influential ones. Therefore, we selected two tRFs&tiRNAs that affect both pathways, tRF-Leu-AAG-001 and tRF-Leu-TAG-015, as candidate markers for follow-up studies.

### Exosomal tRF-Leu-AAG-001 is derived from mast cells in ectopic tissues

**Fig. 3:**
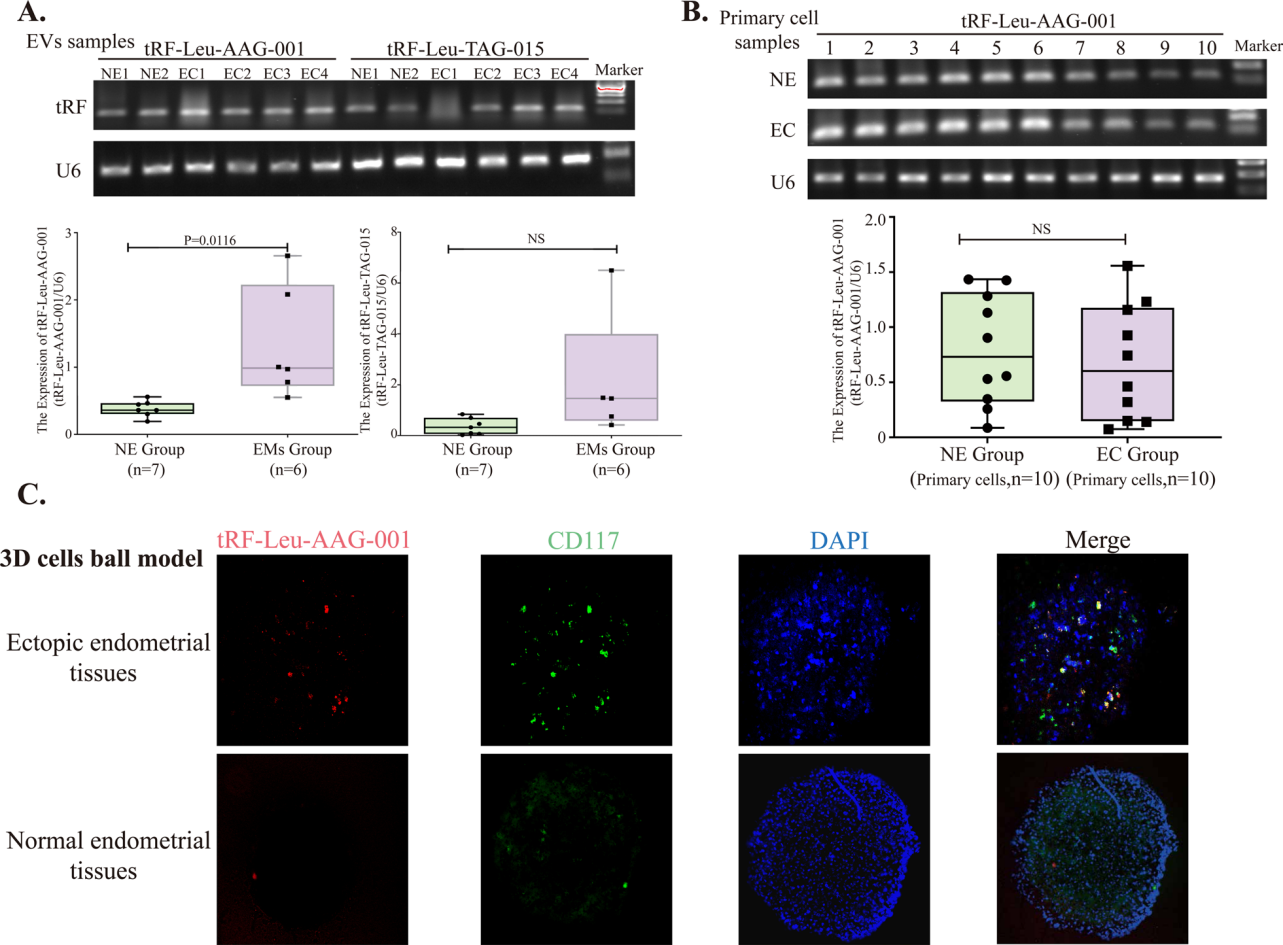
Exosomal tRF-Leu-AAG-001 derived from mast cells in ectopic tissue. **A** Nucleic acid electrophoresis showed that tRF-Leu-AAG-001 was highly expressed in ectopic tissues (*p = 0.0116), while the expression of tRF-Leu-TAG-015 had no statistically difference between the EMs group (n = 6) and the NE group (n = 7). Two-tailed Student’s t-test was used. **B** There was no significant difference in the expression of tRF-Leu-AAG-001 in the primary ectopic endometrial cells (n = 10) and the primary normal endometrial cells (n = 10).Two-tailed Student’s t-test was used. (The original images of the gels were shown in the Additional file 4: Figures). **C** In the 3D cell spheroid model, RNA probes and fluorescence immunolocalization showed that tRF-Leu-AAG-001 was highly expressed in mast cells of the ectopic cell spheroid (200*). Anti-CD117 was the marker of mast cells; DAPI was used for nuclear staining. NE group: normal endometrial group; EC group: ectopic cells

To verify whether tRF is highly expressed in ectopic tissues, we examined the expression of tRF-Leu-AAG-001 and tRF-Leu-TAG-015 in ectopic tissues and normal intimal tissues. The agarose gel electrophoresis result showed that the expression of tRF-Leu-AAG-001 was significantly higher in ectopic tissues (n = 6) than in normal endometrial tissues (n = 7) (*P* = 0.016), while there was no significant difference in the expression of tRF-Leu-TAG-015 between the two groups (Fig. 3A). In order to further explore which cells in the ectopic tissues highly expressed tRF-Leu-AAG-001, we cultured primary ectopic endometrial cells (n = 10) and normal endometrial cells (n = 10) to detect the expression of tRF-Leu-AAG-001 in those two types of endometrial cells. The results pointed out that there was no significant difference in the expression of tRF-Leu-AAG-001 between these two types of endometrial cells (*P* > 0.05) (Fig. 3B). It was considered that tRF-Leu-AAG-001 was mainly enriched in FcεRI signaling pathways. Therefore, we used fluorescence co-localization to detect whether tRF-Leu-AAG-001 was specifically expressed in mast cells. The results showed that tRF-Leu-AAG-001 fluorescent probes were localized in mast cells in the ectopic 3D cell balls while there was almost no expression in the normal endometrial cell spheres. It is therefore suggested that the high expression of tRF-Leu-AAG-001 in ectopic tissues might come from mast cells (Fig. 3C).

### tRF-Leu-AAG-001 regulates inflammatory factors and angiogenesis in mast cell

**Fig. 4:**
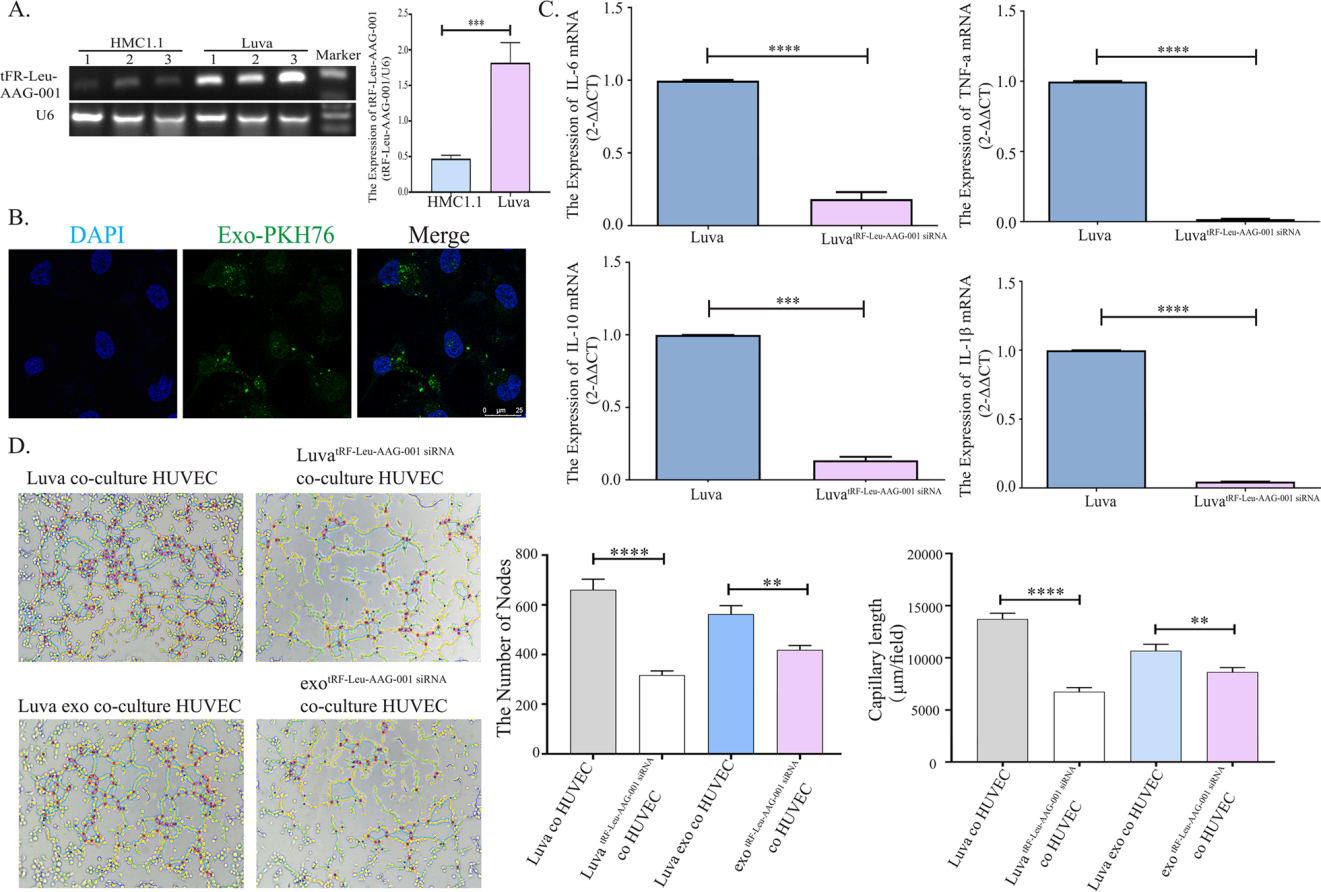
Regulation of inflammatory factors and angiogenesis by tRF-Leu-AAG-001. **A** According to the analysis of gel electrophoresis results, the histogram showed that luva cell line (n = 3) highly expresses tRF-Leu-AAG-001 (***p < 0.001). Two-tailed Student’s t-test was used. (The original images of the gels were shown in the Additional file 4: Figures). **B** Exosomes labeled with PKH67 (Exo-PKH67) were visible in HUVECs after 24 h of incubation. The exosomes were stained green by PKH67 and the nuclei were stained blue by DAPI.Scale bar = 25 μm. **C** The expressions of IL-6, IL-10, IL-1β, and TNF-α were significantly decreased luva cell line (n = 3) after tRF-Leu-AAG-001 was knockdown by siRNA (**** *p* < 0.0001).Two-tailed Student’s t-test was used. **D** The tube formation displayed after exosomal tRF-Leu-AAG-001 derived from luva was able to induce angiogenesis (**p < 0.01, ****p < 0.0001).Two-tailed Student’s t-test was used

Due to the difficulty in the extraction of primary mast cells from ectopic tissues, we used mast cell lines, HMC1.1 and Luva, to instead of primary mast cells. First, we examined the expression of tRF-Leu-AAG-001 in two mast cell lines, the gel electrophoresis result showed that tRF-Leu-AAG-001 was highly expressed in luva (Fig. 4A). We labeled exosomes with PKH67 dye and then added them to cell cultures, and fluorescence images after 24 h showed that exosomes uptake by HUVEC cells (Fig. 4B). We evaluated the mRNA expression of inflammatory factors after tRF-Leu-AAG-001 knockdown in luva and found that the expression of IL-6, IL-10, IL-1β, TNF-α was significantly decreased (Fig. 4C). Meanwhile, we extracted the exosomes derived from luva before and after tRF-Leu-AAG-001 was silenced and co-cultured the exosomes with HUVEC. The results of the tube formation showed that the formation of new blood vessels was markedly reduced after tRF-Leu-AAG-001 knockdown (Fig. 4D).

### Exosomal tRF-Leu-AAG-001 in leucorrhea is correlated with endometriosis

**Fig. 5:**
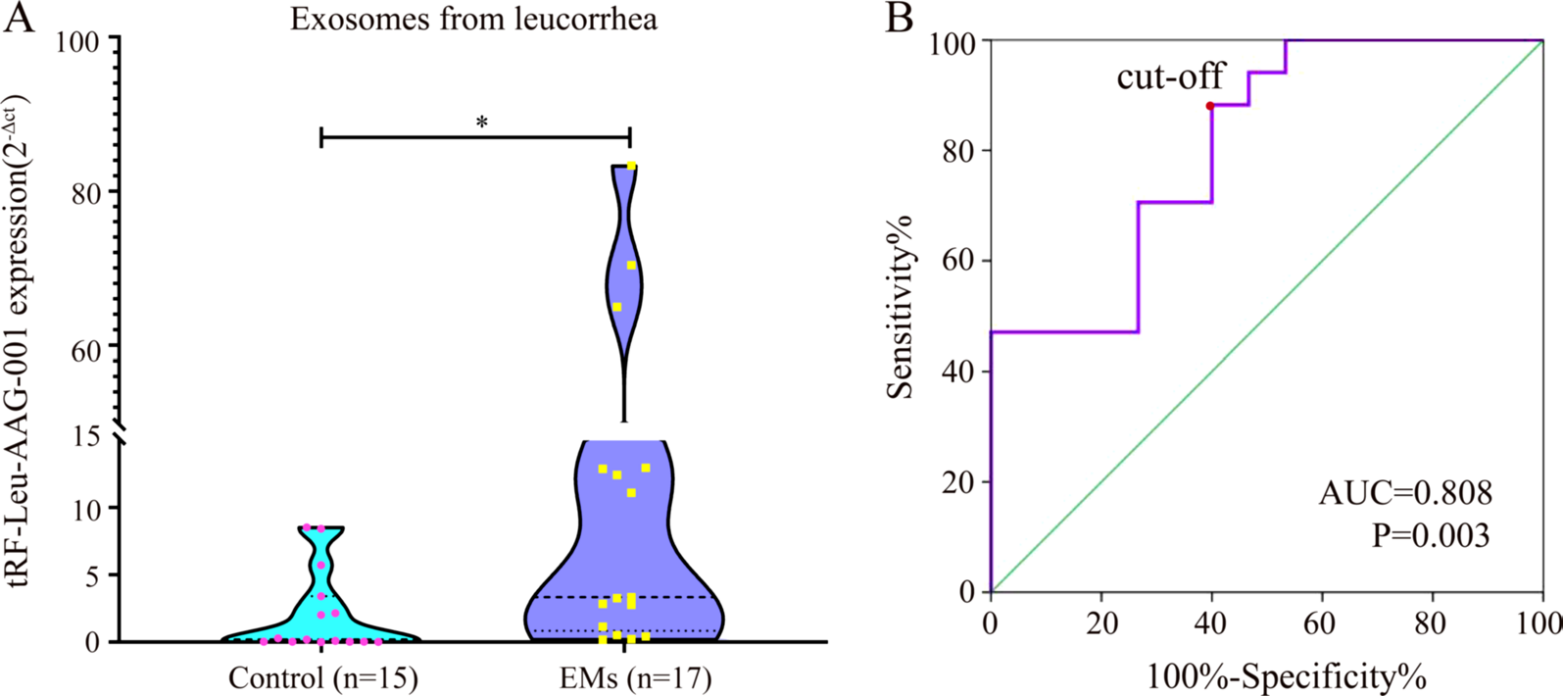
Exosomal tRF-Leu-AAG-001 in leucorrhea is correlated with endometriosis. **A** The violin chart compares the expression of tRF-Leu-AAG-001 in leucorrhea exosomes between the control group (n = 15) and EMs group (n = 17) with statistical significance (*P < 0.05).Mann–Whitney U test was used. **B** The ROC curve of leucorrhea exosomal tRF-Leu-AAG-001, the cutoff value was 0.3513, *p* = 0.003

In order to verify whether exosomal tRF-Leu-AAG-001 can be a marker as a non-invasive diagnosis for endometriosis, we extracted exosomes in the vaginal discharge of patients in the EMs group (n = 17) and the control group (n = 15). qPCR was utilized to quantify the expression of exosomal tRF-Leu-AAG-001, the results manifested the expression of exosomal tRF-Leu-AAG-001 in EMs groups was significantly higher than that in the control group(*p* = 0.0333)(Fig. 5A). The formula's sensitivity and specificity were analyzed to evaluate the occurrence of endometriosis through the receiver operating characteristic (ROC) curve analysis. This analysis revealed that the area under the curve (AUC) was 0.808, the cutoff value was 0.3513, meaning the sensitivity and specificity of exosomal tRF-Leu-AAG-001 were significantly higher (*p* = 0.003) (Fig. 5B). It was suggested that the expression of tRF-Leu-AAG-001 could be used as a potential indicator for the non-invasive diagnosis of endometriosis.

## Discussion

Currently, laparoscopy is still the gold standard for the diagnosis of endometriosis. Although there have been updates on the diagnostic approaches of endometriosis, few studies focus on non-invasive diagnoses. In this study, to find out a potential non-invasive diagnostic marker, we isolated exosomes from ectopic tissue, and the tRNA chip was used to analyze the expression profiles of tRFs & tiRNAs. We obtained 63 tRFs & tiRNAs highly expressed in ectopic tissue-derived exosomes. According to KEGG pathway analysis, tRF-Leu-AAG-001 was selected, which is highly enriched in the VEGF and FcεRI signaling pathway, as a candidate marker. We successfully detected the expression of exosomal tRF-Leu-AAG-001 in the leucorrhea of patients with endometriosis. In addition, we proved that the exosomal tRF-Leu-AAG-001 derived from leucorrhea of EMs patients was a particular indicator. In other diseases, specific tRF&tiRNA have also been gradually considered as reliable biomarkers [27]. In breast cancer, Serum tRF-17-79MP9PP, as a biological marker for detecting breast cancer, has a sensitivity of up to 70% [28]. These altogether supported the great potential of exosomal tRFs as biomarkers in the diagnosis of diseases.

To explore the origin of exosomal tRF-Leu-AAG-001 in ectopic tissue, we assessed the expression of tRF-Leu-AAG-001 in primary ectopic cells. WB results showed no difference in the protein expression level of tRF-Leu-AAG-001 between the EMs group and the control group, although tRF-Leu-AAG-001 was found to be expressed in primary ectopic cells. Subsequently, we analyzed the KEGG pathway and considered the detection of tRF-Leu-AAG-001 in mast cells of ectopic tissue. However, because of the scarcity of mast cells in the ectopic tissue, we established the primary 3D cell sphere model to simulate the physiological environment in humans. We utilized the co-localization of RNA probes and fluorescent antibodies and observed that mast cells in ectopic tissues express a high level of tRF-Leu-AAG-001. Thus, we concluded that the increased expression of exosomal tRF-Leu-AAG-001 in ectopic tissues might be secreted by mast cells.

Mast cells are redient cells and can release abundant cytokines, chemokines, and biologically active mediators [29]. In studies with animals and human tissue, it was found that the numbers of activated mast cells were visibly increased in endometriotic lesions, resulting in inflammation that was caused by mediators and cytokines that were released from activated mast cells [30]. Clinical sample tests demonstrated various cytokines were elevated in the peritoneal fluid of EMs patients, such as IL-6, IL-8, TNF-α, and glycodelin [31, 32]. In porcine and rabbit, EMs models have supported the concept of a central role for mast cells in a “nerve-mast cell-myofibroblast axis” in some inflammatory processes [33]. In addition, there are reports suggested that owing to the specific tryptases and chymases, mast cells exist in ectopic lesions shows the same angiogenic function as macrophages and fibroblasts [34, 35]. However, only a few studies have explored why mast cells become “excited” in ectopic foci. In our experiment, high expression of tRF-Leu-AAG-001 in mast cells in the ectopic foci triggered the mast cells to express more inflammatory factors IL-6, IL-10, IL-1β, TNF-α. It was implied that mast cells were involved in the occurrence of inflammation in ectopic foci. In addition, as the VEGF signaling pathway was also an enrichment pathway for tRF-Leu-AAG-001, we verified that the exosomal tRF-Leu-AAG-001 secreted by mast cells was capable of promoting the formation of peripheral blood vessels. These results provide a reasonable explanation for the abnormal biological function of mast cells in endometriosis.

In summary, we investigated the biological functions of tRF-Leu-AAG-001 in mast cells and its secreted exosomal tRF-Leu-AAG-001 in ectopic tissues. It was concluded that tRF-Leu-AAG-001 in mast cells had a significant role in promoting inflammation and angiogenesis in EMs. However, we have not deeply studied the molecular mechanism of the abnormal increase of tRF-Leu-AAG-001 in mast cells. At the same time, it cannot be denied that although mast cells are one of the members of antigen-presenting cells, their number in ectopic tissues is incomparable to other APCs. Therefore, additional research needs to be done to examine whether mast cell tRF-Leu-AAG-001 can play a crucial role in the pathological process of EMs in the future. Secondly, we used optimized technology to detect and evaluate the exosomes tRF-Leu-AAG-001 in leucorrhea with EMs. Overall, these results indicated that leucorrhea exosomal tRF-Leu-AAG-001 has high specificity and sensitivity for the differential diagnosis of EMs. Future experiments will be done on more clinical samples to support exosomal tRF-Leu-AAG-001 is reliable as a non-invasive diagnostic marker (Additional file 3).

## Supplementary Information

**Additional file 1** Subject's Characteristics in women with and withoutendometriosis.

**Additional file 2** 7 up regulated exosomal tRFs and tiRNAsof EMs.

**Additional file 3** Western blot results of exosomal marker protein Flotillion 1,HSP70 ,CD63 ,Calnexin.

**Additional file 4** The original images of the gelsand blots.

## Data Availability

The datasets generated and analysed during the current study are available in the GEO DataSets repository, https://www.ncbi.nlm.nih.gov/geo/query/acc.cgi?acc=GSE185273.

## Abbreviations

Ems: Endometriosis
tRFs: TRNA-derived fragments
tsRNAs: TRNA-derived small RNAs
tiRNAs: TRNA halves
EVs: Extracellular vesicles
HUVECs: Human umbilical vein endothelial cells
TME: Transmission electron microscopy
NTA: Nanoparticle tracking analysis
