# Supplementary material for: Exosomal tRF-Leu-AAG-001 derived from mast cell as a potential non-invasive diagnostic biomarker for endometriosis
Source: BMC Womens Health. 2022 Jun 25;22:253. doi: 10.1186/s12905-022-01827-6 (PMC9233364; doi:10.1186/s12905-022-01827-6)
Supplement: Supplementary file 1 — Additional file 1 Subject's Characteristics in women with and withoutendometriosis. [file 12905_2022_1827_MOESM1_ESM.docx]

**Supplementary Table1** | Subject’s Characteristics in women with and without endometriosis

| **Parameters** | **Endometriosis (n=26）** | **No Endometriosis (n=25)** | **P value** |
| --- | --- | --- | --- |
| **Age** | 34.8±7.9 | 38.7±10.1 | 0.2316 |
| **Sample type** |  |  |  |
| leucorrhea | 17(65.38%) | 15(60.00%) |  |
| endometrial tissue | 9(34.62%) | 10(40.00%) |  |
| **Dysmenorrhea** | 14(53.85%) | 3(12.00%) |  |
| **Menstrual cycle phase** |  |  |  |
| Proliferative | 14(53.85%) | 13(52.00%) |  |
| Secretory | 12(46.15%) | 12(48.00%) |  |
| **Ovarian endometriosis involving** |  |  |  |
| Pelvic | 21(80.77%) |  |  |
| Retroperitoneal | 5(19.23%) |  |  |
| The uterosacral ligament | 8(30.77%) |  |  |
| Ureteral | 4(11.53%) |  |  |
| **Size of Cyst(cm^3^)** |  |  |  |
| <64cm^3^ | 8(30.77%) |  |  |
| <216cm^3^,≥64cm^3^ | 13(53.85%) |  |  |
| ≥216cm^3^ | 5(19.23%) |  |  |
