# Supplementary material for: Exosomal tRF-Leu-AAG-001 derived from mast cell as a potential non-invasive diagnostic biomarker for endometriosis
Source: BMC Womens Health. 2022 Jun 25;22:253. doi: 10.1186/s12905-022-01827-6 (PMC9233364; doi:10.1186/s12905-022-01827-6)
Supplement: Supplementary file 2 — Additional file 2 7 up regulated exosomal tRFs and tiRNAsof EMs. [file 12905_2022_1827_MOESM2_ESM.docx]

**Supplementary Table2 |** 7 up-regulated exosomal tRFs and tiRNAs of EMs

| **tRF_ID** | **tRF_Seq** | **Type** | **tRFdb_ID** | **MINTbase_ID** | **log2FC** | **Fold_Change** | **p_value** |
| --- | --- | --- | --- | --- | --- | --- | --- |
| tiRNA-Leu-CAG-001 | GTCAGGATGGCCGAGCGGTCTAAGGCGCTGCGTTC | tiRNA-5 | - | tRF-35-SP5830MMUKLYM9 | 8.523441113 | 367.969177 | 1.05399E-05 |
| tRF-Leu-TAG-015 | ATCCCACCACTGCCACCA | tRF-3a | 3010a | tRF-18-HR05X6D2 | 7.956890623 | 248.4635864 | 5.58058E-05 |
| tiRNA-Gly-CCC-003 | GCGCCGCTGGTGTAGTGGTATCATGCAAGATTC | tiRNA-5 | - | tRF-33-Q1Q89P9L842205 | 4.982746957 | 31.61959441 | 1.37402E-05 |
| tRF-Leu-AAG-001 | ATCCCACCGCTGCCACCA | tRF-3a | 3001a | tRF-18-HR0VX6D2 | 4.557579655 | 23.54876761 | 0.000140686 |
| tRF-Leu-TAA-005 | ACCAGGATGGCCGAGT | tRF-5a | - | - | 4.232721684 | 18.80079397 | 0.001276811 |
| tiRNA-Lys-CTT-003 | GCCCGGCTAGCTCAGTCGGTAGAGCATGGGACTC | tiRNA-5 | - | tRF-34-PSQP4PW3FJIKE5 | 3.783076071 | 13.76636795 | 0.000400105 |
| tiRNA-Val-CAC-002 | GTTTCCGTAGTGTAGCGGTTATCACATTCGCCTC | tiRNA-5 | - | tRF-34-79MP9PMNH5IS15 | 2.773041768 | 6.835475822 | 0.011965883 |

log2FC(EMs vs. Control group)
