# Supplementary figures and images for: Exosomal tRF-Leu-AAG-001 derived from mast cell as a potential non-invasive diagnostic biomarker for endometriosis
Source: BMC Womens Health. 2022 Jun 25;22:253. doi: 10.1186/s12905-022-01827-6 (PMC9233364; doi:10.1186/s12905-022-01827-6)

## HSP70

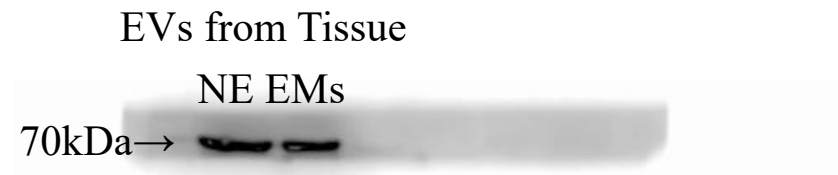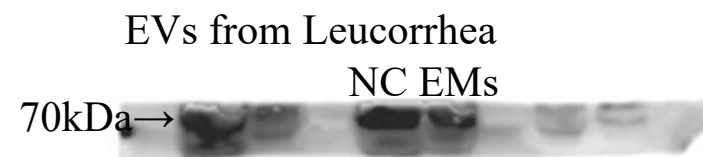

## Flotillin1

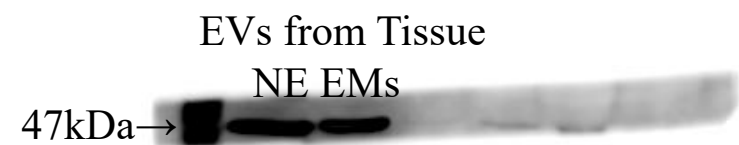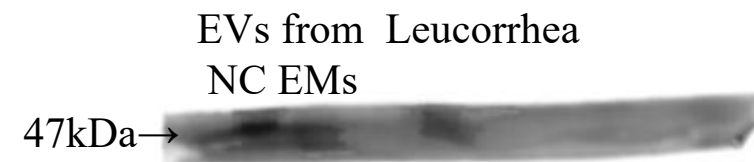

**CD63**

EVs from  
Tissue   Leucorrhea  
NE   EMs   NC   EMs

25kDa→

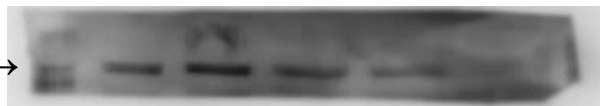

**Calnexin**

Cell   EVs

75kDa→

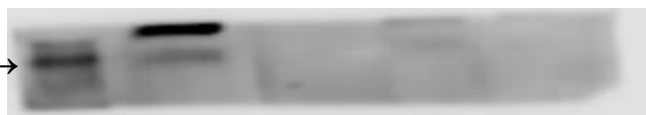

Supplement: Supplementary file 3 — Additional file 3 Western blot results of exosomal marker protein Flotillion 1,HSP70 ,CD63 ,Calnexin. [file 12905_2022_1827_MOESM3_ESM.pdf]
