# Supplementary material for: Exosomal tRF-Leu-AAG-001 derived from mast cell as a potential non-invasive diagnostic biomarker for endometriosis
Source: BMC Womens Health. 2022 Jun 25;22:253. doi: 10.1186/s12905-022-01827-6 (PMC9233364; doi:10.1186/s12905-022-01827-6)

Supplementary figures

Figure 1S(C) | The original images of exosomal marker protein HSP70.

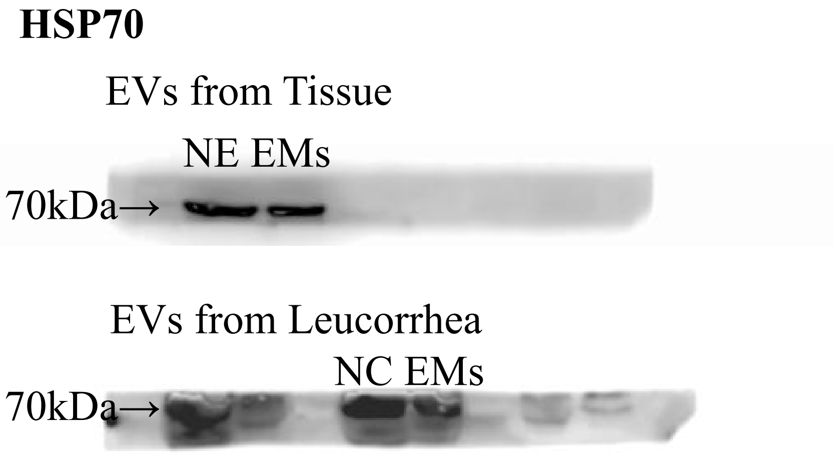

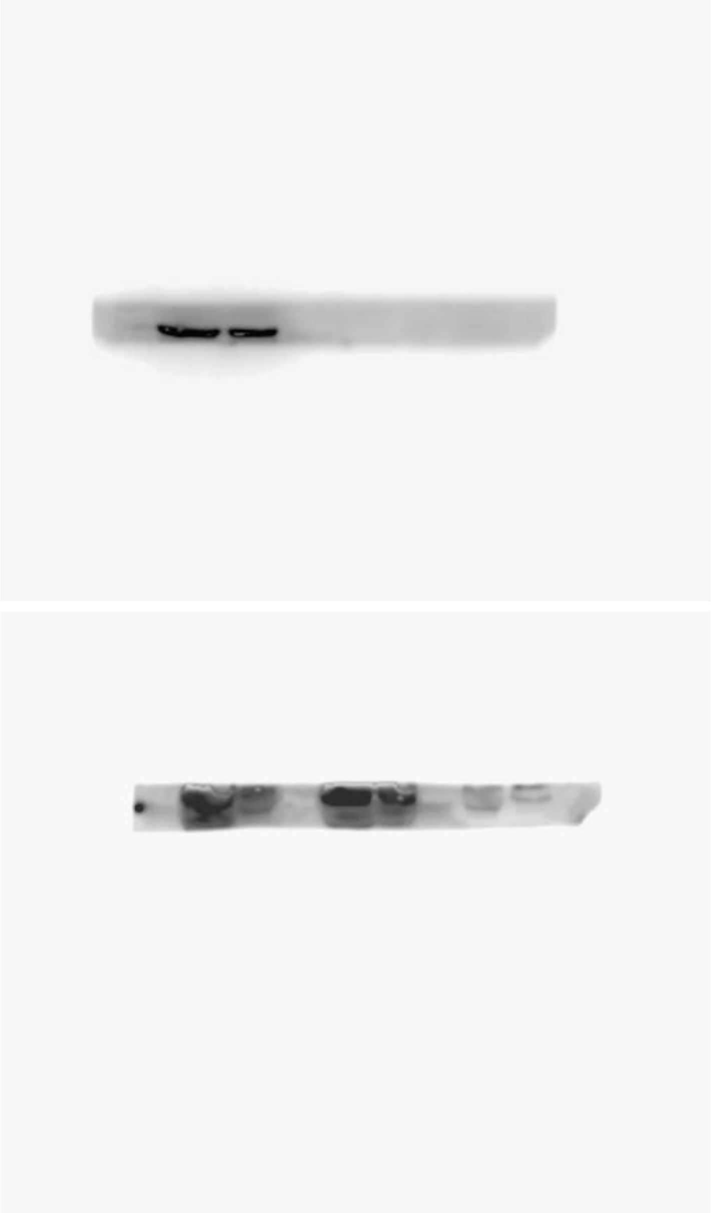

Figure 1S(C) **|** The original images of exosomal marker protein Flotillin1.

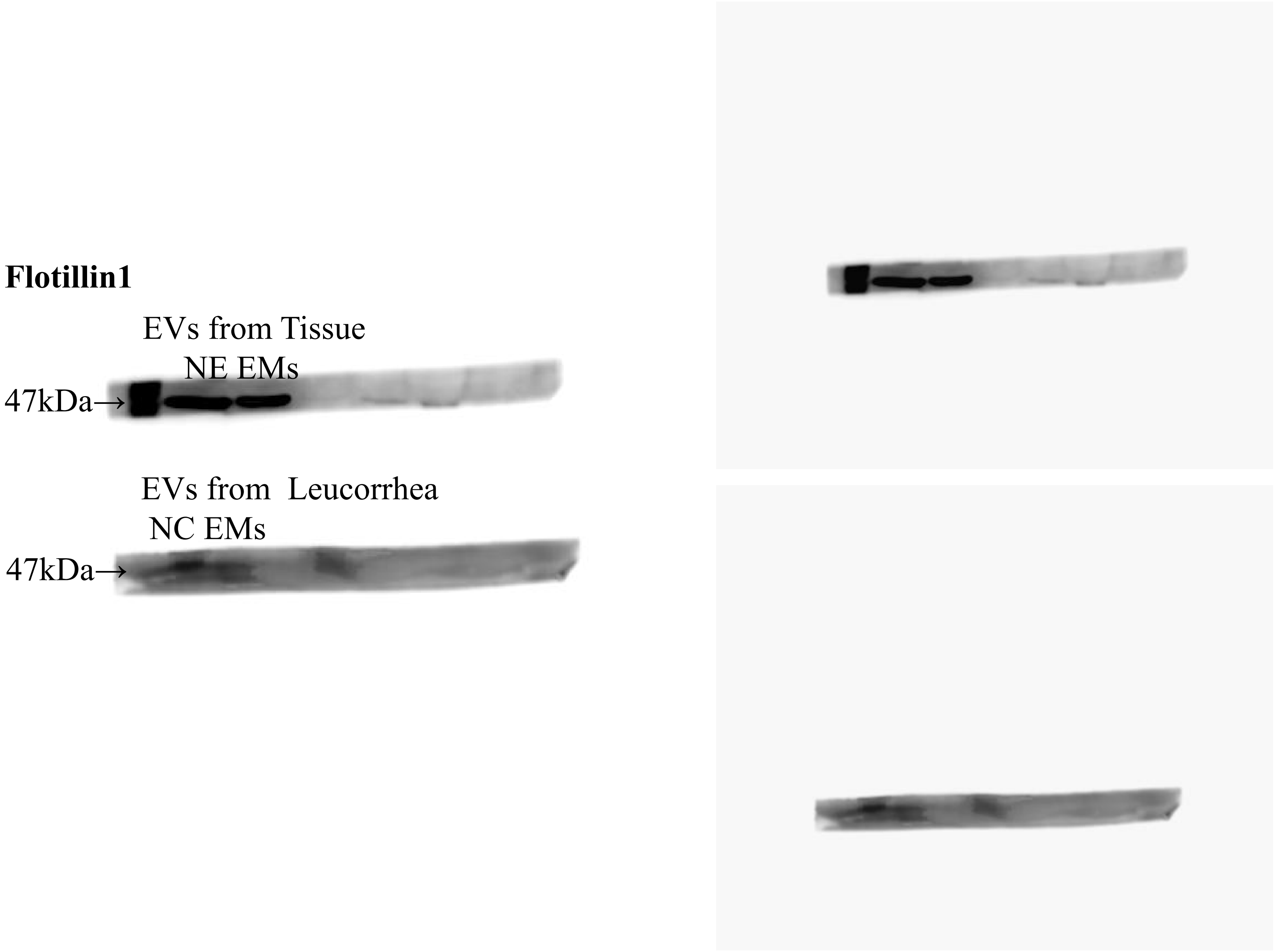

**Figure 1S(C) | **The original images of exosomal marker protein CD63 and Calnexin.

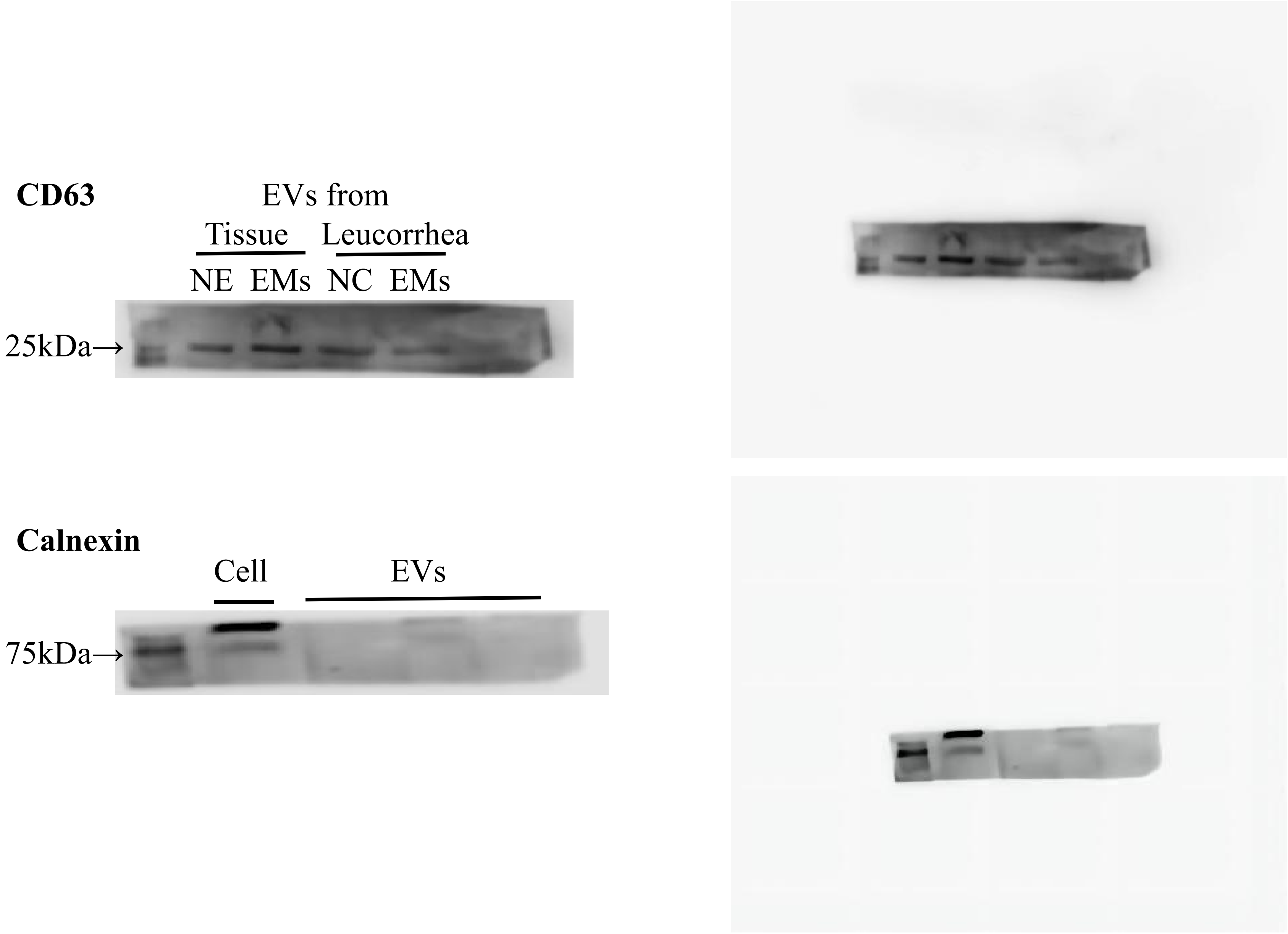

Figure 3S(A) | The expression of tRF (left) and U6 (right) in ectopic tissues.

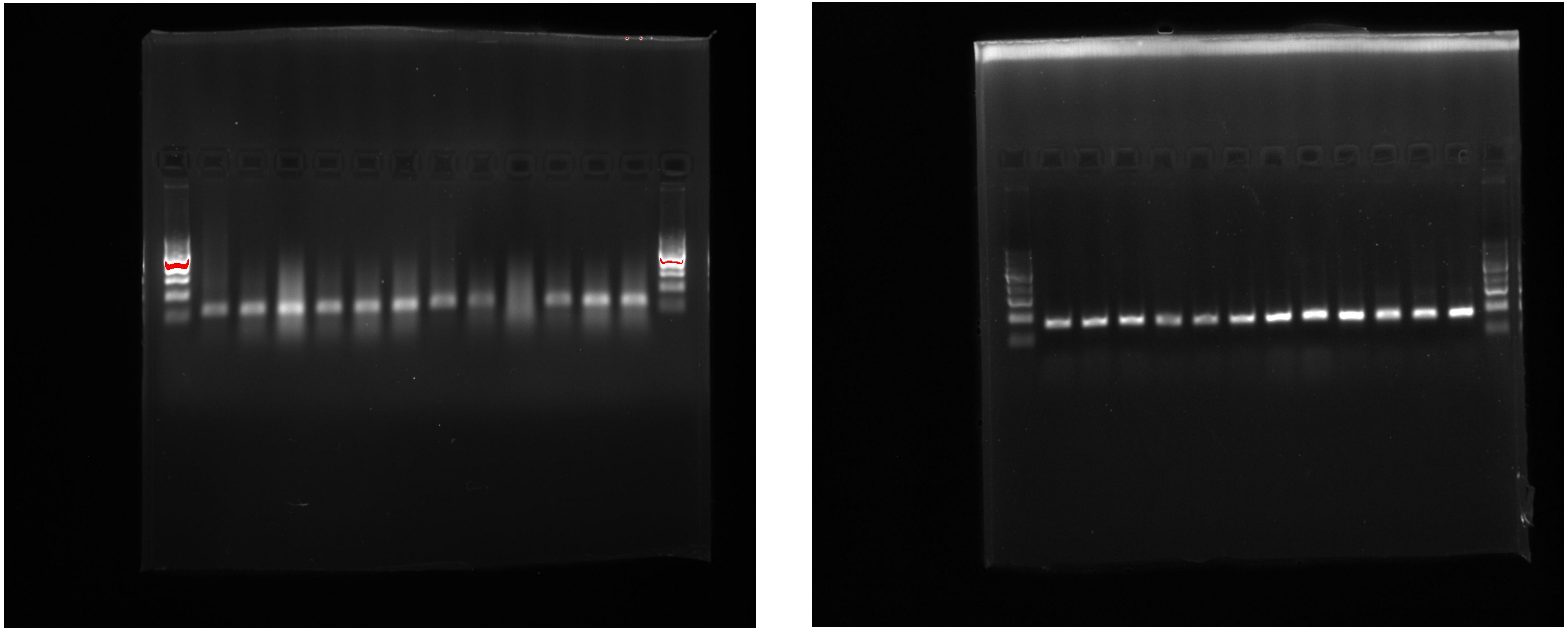

Figure 3S(B) | The expression of tRF-Leu-AAG-001 in normal endometrial tissues (a) and ectopic tissues (b). The expression of u6 (c).

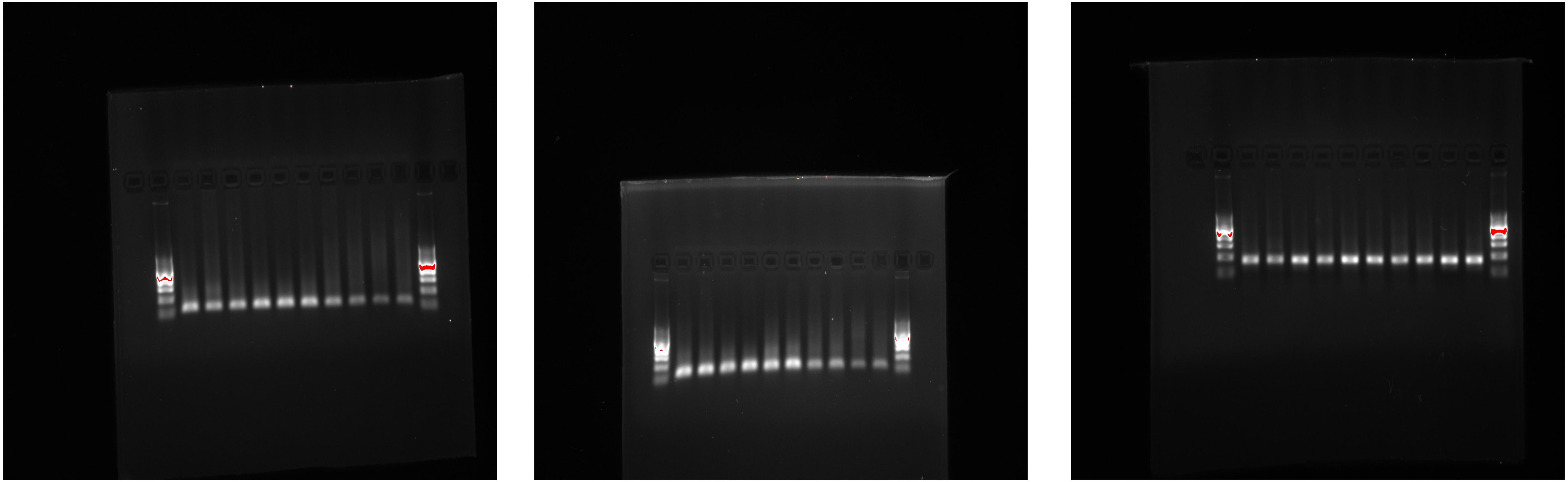

(a) (b) (c)

Figure 4S(A) | The expression of tRF-Leu-AAG-001 (left) and u6 (right) in two mast cell lines.

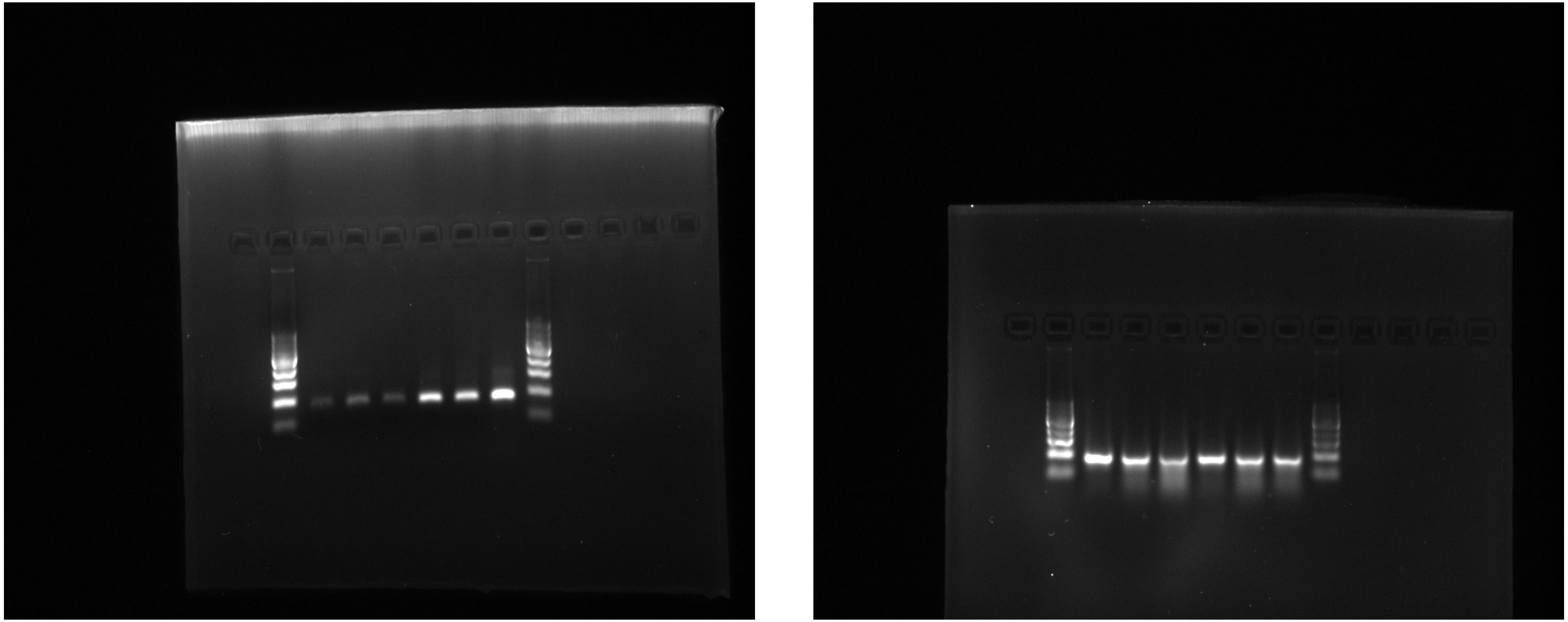

Supplement: Supplementary file 4 — Additional file 4 The original images of the gelsand blots. [file 12905_2022_1827_MOESM4_ESM.pdf]
